# Antibody and T cell responses against wild-type and Omicron SARS-CoV-2 after third-dose BNT162b2 in adolescents

**DOI:** 10.1038/s41392-022-01282-7

**Published:** 2022-12-14

**Authors:** Xiaofeng Mu, Carolyn A. Cohen, Daniel Leung, Jaime S. Rosa Duque, Samuel M. S. Cheng, Yuet Chung, Howard H. W. Wong, Amos M. T. Lee, Wing Yan Li, Issan Y. S. Tam, Jennifer H. Y. Lam, Derek H. L. Lee, Sau Man Chan, Leo C. H. Tsang, Karl C. K. Chan, John K. C. Li, Leo L. H. Luk, Sara Chaothai, Kelvin K. H. Kwan, Nym Coco Chu, Masashi Mori, Trushar Jeevan, Ahmed Kandeil, Richard J. Webby, Wenwei Tu, Sophie A. Valkenburg, Malik Peiris, Yu Lung Lau

**Affiliations:** 1 Department of Paediatrics and Adolescent Medicine, The University of Hong Kong, Hong Kong, China; 2 HKU-Pasteur Research Pole, School of Public Health, The University of Hong Kong, Hong Kong, China; 3 School of Public Health, The University of Hong Kong, Hong Kong, China; 4 Research Institute for Bioresources and Biotechnology, Ishikawa Prefectural University, Nonoichi, Japan; 5 Department of Infectious Diseases, St Jude Children’s Research Hospital, Memphis, TN USA; 6 Department of Microbiology and Immunology, Peter Doherty Institute for Infection and Immunity, University of Melbourne, Melbourne, VIC Australia; 7 Centre for Immunology & Infection C2i, Hong Kong, China

**Keywords:** Vaccines, Adaptive immunity

## Abstract

The high effectiveness of the third dose of BNT162b2 in healthy adolescents against Omicron BA.1 has been reported in some studies, but immune responses conferring this protection are not yet elucidated. In this analysis, our study (NCT04800133) aims to evaluate the humoral and cellular responses against wild-type and Omicron (BA.1, BA.2 and/or BA.5) SARS-CoV-2 before and after a third dose of BNT162b2 in healthy adolescents. At 5 months after 2 doses, S IgG, S IgG Fc receptor-binding, and neutralising antibody responses waned significantly, yet neutralising antibodies remained detectable in all tested adolescents and S IgG avidity increased from 1 month after 2 doses. The antibody responses and S-specific IFN-γ^+^ and IL-2^+^ CD8^+^ T cell responses were significantly boosted in healthy adolescents after a homologous third dose of BNT162b2. Compared to adults, humoral responses for the third dose were non-inferior or superior in adolescents. The S-specific IFN-γ^+^ and IL-2^+^ CD4^+^ and CD8^+^ T cell responses in adolescents and adults were comparable or non-inferior. Interestingly, after 3 doses, adolescents had preserved S IgG, S IgG avidity, S IgG FcγRIIIa-binding, against Omicron BA.2, as well as preserved cellular responses against BA.1 S and moderate neutralisation levels against BA.1, BA.2 and BA.5. Sera from 100 and 96% of adolescents tested at 1 and 5 months after two doses could also neutralise BA.1. Our study found high antibody and T cell responses, including potent cross-variant reactivity, after three doses of BNT162b2 vaccine in adolescents in its current formulation, suggesting that current vaccines can be protective against symptomatic Omicron disease.

## Introduction

Unvaccinated children and adolescents have a high risk of SARS-CoV-2 infection and it may be associated with hospitalisations, multi-system inflammatory syndrome and long COVID.^1–3^ As one of the two most used vaccines worldwide, Pfizer-BioNTech-Fosun Pharma COVID-19 (BNT162b2) vaccine is a nucleoside-modified and lipid nanoparticle-formulated mRNA vaccine encoding the wild-type SARS-CoV-2 spike (S) glycoprotein,^4^ which has demonstrated 95% efficacy in preventing COVID-19 after two doses in adults.^5^ The Food and Drug Administration (FDA) issued an Emergency Use Authorization (EUA) for the use of BNT162b2 in adolescents aged 12–15 years on May 10, 2021.^6^ In a phase 3 study, the efficacy of two-dose BNT162b2 was 100% in adolescents aged 12–15 years.^7^ Our previous data also showed significantly higher humoral responses, including total S IgG, virus neutralisation, S IgG avidity and Fcγ receptor-binding antibody responses in adolescents aged 11–17 years after two doses of BNT162b2 than two doses of CoronaVac.^8^

Vaccine effectiveness (VE) has been found to decline at 6 months after the second dose of the BNT162b2 vaccine in adults^9,10^ and adolescents.^11^ VE was also reduced during periods predominated by Omicron BA.1, which contains more than 30 mutations in its S protein, enabling dramatic neutralisation escape.^12,13^ Further Omicron sublineages have emerged, with a BA.2 epidemic wave affecting Hong Kong in January 2022, whilst BA.5 has become predominant worldwide since July 2022. In the UK, VE at 2 weeks after two doses of BNT162b2 vaccine declined to 65.5% in adults,^14^ and 83.1% in adolescents aged 12–15 years,^15^ respectively, for Omicron BA.1. Waning VE against variants of concern was boosted by a booster dose in adults. In England, a real-world study showed 95% VE against symptomatic disease, and around 97–99% against hospitalisation or death at 14–34 days after a third dose of BNT162b2 in adults.^16^ In Israel, the third dose of BNT162b2 had 95.3% efficacy against symptomatic COVID-19.^17^ A homologous third dose of BNT162b2 increased VE against symptomatic COVID to 67.2% in adults during BA.1 predominance.^14^ It was hypothesised that a third dose of BNT162b2 in adolescents would further protect against Omicron BA.1 infection. In the United States, Klein et al. found 81% VE against the emergency department and urgent care encounters in adolescents aged 16–17 years who received three doses of BNT162b2 during the BA.1 wave.^18^ Yet, little is known about the humoral or cellular immune responses after three doses of BNT162b2 in healthy adolescents.

Antibody responses have been found to correlate with vaccine efficacy against symptomatic COVID-19.^19^ Apart from antibody responses, CD8^+^ cytotoxic T lymphocytes (CTLs) can eliminate virus-infected cells directly and differentiated CD4^+^ T helper cells can coordinate a virus-specific immune response.^20,21^ Robust memory CD8^+^ and CD4^+^ T cells may provide long-lasting immunity against SARS-CoV-2 even in the absence of antibody responses and the neutralising antibody escape by variants like Omicron.^22–24^ Circulating effector T cells responses to the Omicron variants were preserved both in prior infected patients and vaccinated individuals.^12^ However, binding and neutralising antibody and T cell responses against Omicron variants after the third dose of the BNT162b2 vaccine in adolescents remain unknown.

Following our previous study, here we evaluated both humoral responses against the wild-type (WT) and Omicron BA.1, BA.2 and/or BA.5, including antibody binding and neutralising functions, with ELISA-based assays and authentic plaque reduction neutralisation test, and cellular responses against the WT and Omicron BA.1 by detection of intracellular IFN-γ^+^ and IL-2^+^ CD4^+^ and CD8^+^ T cells by flow cytometry, before and after the third dose of BNT162b2 in healthy adolescents aged 11–17 years compared to that in healthy adults.

## Results

### Enrolment of study participants

Fifty healthy adolescents aged 11–17 years and 80 healthy adults aged 18 years or older received a third dose of BNT162b2 by February 27, 2022 in our study (Supplementary Fig. 1a). Excluding participants who were infected during the study as determined by the presence of ORF8 antibodies^25^ or contributed no safety data and did not attend follow-up clinic, 28 adolescents aged 11–17 years (mean 13.7 years old) and 41 adults aged 18 years or above (mean 48.4 years old) were included in healthy expanded analysis population, with comparable sex and ethnicity distribution (Supplementary Table 1). Primary immunogenicity was assessed in the evaluable analysis population, which included participants with valid and timely immunogenicity results and no protocol deviations. Immunogenicity analyses were repeated in the expanded analysis population with relaxed intervals for vaccination and blood sampling to further confirm the findings. The expanded analysis population in this study received dose 3 at least 56 days (whereas it was 84 days in the evaluable analysis population) after dose 1 and had a valid and determinate relevant immunogenicity result for the particular analysis from a blood sample taken between 6-56 days post-dose 3 (13–42 days post-dose 3 in the evaluable analysis population) and before any further doses. Doses 1 and 2 were given 21–28 days apart. In evaluable analysis populations (adolescents *N* = 28, adults *N* = 33), bloods were collected 1 month (28.5 days) after dose 2 (post-dose 2), 5 months (155 days) after dose 2 (pre-dose 3), and 3 weeks (22.7 days) after booster (post-dose 3), as shown in Supplementary Fig. 1b. In expanded analysis populations (adolescents *N* = 28, adults *N* = 41), bloods were collected on average 31 days after dose 2 (post-dose 2), 160 days after dose 2 (pre-dose 3), and 25 days after dose 3 (post-dose 3). The protocol and statistical analysis plan are available in the Supplementary materials.

### Adolescent humoral immune responses are boosted and non-inferior to adults

For the primary humoral immunogenicity analysis, sera from evaluable adolescents and adults were collected, and antibody responses against the WT virus with SARS-CoV-2 Spike (S) IgG, S receptor-binding domain (S-RBD) IgG, S IgG avidity, and S Fcγ receptor III-a (FcγRIIIa)-binding were tested by enzyme-linked immunosorbent assay (ELISA). ACE2-blocking antibody was estimated by surrogate virus neutralisation test (sVNT). A plaque reduction neutralisation test (PRNT) was also performed.

**Fig. 1:**
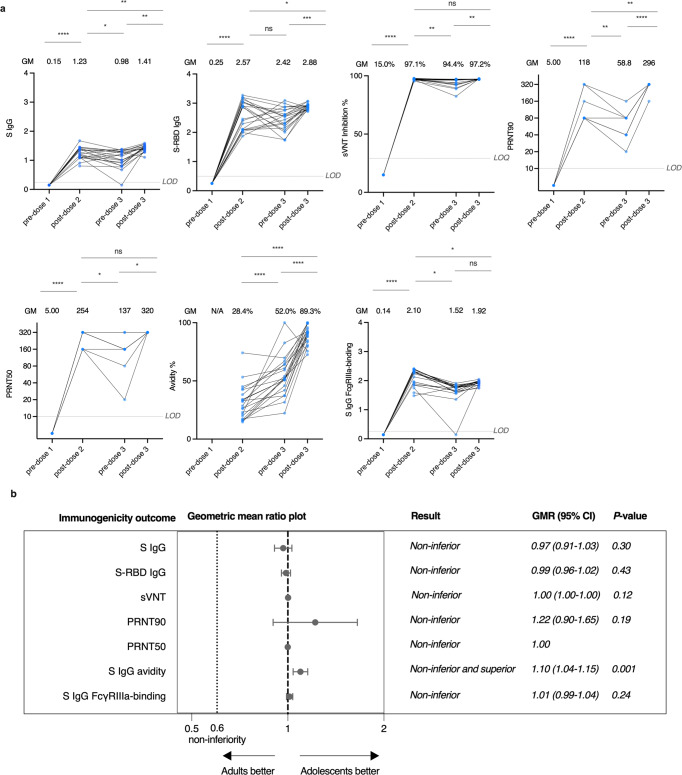
Adolescents have boosted and non-inferior humoral immune responses to the WT virus in comparison to adults after the third dose of the BNT162b2 vaccine. Humoral responses were compared at pre-dose 1, post-dose 2 (1 month after dose 2), pre-dose 3 (5 months after dose 2) and post-dose 3 (3 weeks after booster) in evaluable adolescents. **a** Longitudinal analysis of S IgG (pre-dose 1 *N* = 12, post-dose 2 *N* = 21, pre-dose 3 *N* = 21, post-dose 3 *N* = 21), S-RBD IgG (pre-dose 1 *N* = 21, post-dose 2 *N* = 21, pre-dose 3 *N* = 21, post-dose 3 *N* = 21), sVNT inhibition (pre-dose 1 *N* = 21, post-dose 2 *N* = 21, pre-dose 3 *N* = 21, post-dose 3 *N* = 18), PRNT90 (pre-dose 1 *N* = 9, post-dose 2 *N* = 9, pre-dose 3 *N* = 9, post-dose 3 *N* = 9), PRNT50 (pre-dose 1 *N* = 9, post-dose 2 *N* = 9, pre-dose 3 *N* = 9, post-dose 3 *N* = 9), S IgG avidity (pre-dose 1 *N* = 0, post-dose 2 *N* = 21, pre-dose 3 *N* = 20, post-dose 3 *N* = 21) and S IgG FcγRIIIa-binding (pre-dose 1 *N* = 12, post-dose 2 *N* = 21, pre-dose 3 *N* = 21, post-dose 3 *N* = 21) in evaluable adolescents. A third dose booster increased humoral responses except for S IgG FcγRIIIa-binding. Importantly, S IgG, S-RBD IgG, PRNT50 and S IgG avidity were higher post-dose 3 compared to post-dose 2, while there was a reduction in S IgG FcγRIIIa-binding. Longitudinal analysis was determined using paired *t*-test after natural logarithmic transformation with *p* values denoted. Dots representing the same participants are linked by a straight line. Data labels and centre lines show geometric means (GM) estimates, with corresponding 95% confidence intervals shown by error bars. **b** Non-inferiority testing of S IgG (adolescent *N* = 28, adult *N* = 28), S-RBD IgG (adolescent *N* = 28, adult *N* = 33), sVNT inhibition (adolescent *N* = 25, adult *N* = 33), PRNT90 (adolescent *N* = 14, adult *N* = 14), PRNT50 (adolescent *N* = 14, adult *N* = 14), S IgG avidity (adolescent *N* = 28, adult *N* = 28) and S IgG FcγRIIIa-binding (adolescent *N* = 28, adult *N* = 28) in evaluable adolescents in comparison to adults. These humoral responses were non-inferior, except for S IgG avidity, which was non-inferior and superior. Geometric mean ratios and their associated two-sided 95% confidence intervals (CIs) were plotted. There was no associated 95% CI for PRNT50, as all values in both groups were equal, at the upper LOD of 320. *p < 0.05, **p < 0.01, ***p < 0.001, ****p < 0.0001. ns not significant. LOD limits of detection, LOQ limits of quantification, WT wild-type, S-RBD spike-receptor-binding domain, sVNT surrogate virus neutralisation test, PRNT plaque reduction neutralisation test, FcγRIIIa Fcγ receptor IIIa

To investigate the durability of antibody responses in evaluable adolescents, the tests were performed at all timepoints, including pre-dose 1, post-dose 2, pre-dose 3 and post-dose 3. An interim analysis of immunogenicity post-dose 2 has been previously performed.^8^ Most humoral responses moderately declined at pre-dose 3 when compared with that at post-dose 2, but significantly increased at post-dose 3, including S IgG [geometric mean (GM)-optical density-450 (OD450) post-dose 2, 1.23 vs pre-dose 3, 0.98 vs post-dose 3, 1.41], sVNT (GM-% inhibition 97.1 vs 94.4 vs 97.2%), PRNT90 (GM-PRNT90 118 vs 58.8 vs 296) and PRNT50 (GM-PRNT50 254 vs 137 vs 320) (Fig. 1a). Interestingly, S-RBD IgG did not decrease from post-dose 2 to pre-dose 3 (GM-OD450 2.57 vs 2.42). S IgG avidity index increased continually from post-dose 2 to post-dose 3 (GM avidity % 28.4 vs 52.0 vs 89.3%) (Fig. 1a). S IgG FcγRIIIa-binding were not significantly boosted by dose 3 (GM-OD450 2.10 vs 1.52 vs 1.92) (Fig. 1a).

We studied antibody and T cell responses to the third dose in adolescents and adults, and high antibody responses were found in both evaluable and expanded adolescents and adults (Table 1 and Supplementary Table 2). We tested whether the third dose was non-inferior in adolescents compared to adults, by calculating their geometric mean ratios (GMRs) and 95% confidence intervals (CI) of various immunogenicity outcomes, and assessed whether the humoral responses to the WT virus in adolescents were non-inferior to those in adults by the same methods as our previous study.^8^ Compared to adults, humoral responses including neutralising and binding antibodies were all non-inferior, or even superior, in evaluable adolescents after the third dose as measured by S IgG (GM-OD450 1.44 vs 1.39, GMR 0.97, 95% CI 0.91–1.03), S-RBD IgG (GM-OD450 2.93 vs 2.89, GMR 0.99, 95% CI 0.96–1.02), sVNT (GM % inhibition 97.0 vs 97.1, GMR 1.00, 95% CI 1.00–1.00), PRNT90 (GM-PRNT90 285 vs 296, GMR 1.22, 95% CI 0.90–1.65), PRNT50 (GM-PRNT50 320 vs 320, GMR 1.00, 95% CI not applicable as all individual values were 320), S IgG avidity (GM-% avidity 81.0 vs 88.8%, GMR 1.10, 95% CI 1.04–1.15), and S IgG FcγRIIIa-binding (GM-OD450 1.87 vs 1.90, GMR 1.01, 95% CI 0.99–1.04) (Fig. 1b and Supplementary Fig. 2a). Antibody responses were further confirmed in the expanded analysis population and similar results were found (Supplementary Fig. 2b). These results indicate that the third dose of BNT162b2 induces high levels of humoral responses against the WT virus in adolescents, which are comparable to that in adults.

**Table 1:** Humoral immunogenicity outcomes against wild-type SARS-CoV-2 after the third dose of BNT162b2 in the evaluable analysis population

	Adolescents three doses	Adults three doses
**S IgG on ELISA**		
*N*	28	28
GM-OD450 value (95% CI)	1.39 (1.32–1.48)	1.44 (1.40–1.48)
% positive (≥LOD at 0.3)	100%, *P* > 0.9999	100%
**S-RBD IgG on ELISA**		
*N*	28	33
GM-OD450 value (95% CI)	2.89 (2.85–2.93)	2.93 (2.85–3.01)
% positive (≥LOD at 0.5)	100%, *P* > 0.9999	100%
**S-RBD ACE2-blocking antibody on sVNT**		
*N*	25	33
GM % inhibition (95% CI)	97.1% (97.0–97.2%)	97.0% (96.9–97.1%)
% positive (≥LOQ at 30%)	100%*, P* > 0.9999	100%
**Neutralising antibody on PRNT**		
*N*	14	14
GM-PRNT90 (95% CI)	263 (218–317)	215 (166–279)
% positive (≥LOD at 10)	100%, *P* > 0.9999	100%
GM-PRNT50 (95% CI)	320 (320–320)	320 (320–320)
% positive (≥LOD at 10)	100%, *P* > 0.9999	100%
**S IgG avidity on ELISA**		
*N*	28	28
GM avidity index (95% CI)	88.8% (85.8.8–91.9)	81.0% (77.7–84.4)
**S IgG FcγRIIIa-binding on ELISA**		
*N*	28	28
GM-OD450 value (95% CI)	1.90 (1.86–1.93%)	1.87 (1.85–1.89%)
% positive (≥LOD at 0.28)	100%, >0.9999	100%

*P* values compare the proportion of positive responses between adolescents and adults by Fisher’s exact test

*S* spike protein, *ELISA* enzyme-linked immunosorbent assay, GM geometric mean, *OD* optical density, *LOD* limit of detection, *LOQ* limit of quantification, *CI* confidence interval, *RBD* receptor-binding domain, *ACE-2* angiotensin-converting enzyme-2, *sVNT* surrogate virus neutralisation test, *PRNT* plaque reduction neutralisation test, *PRNT90* 90% plaque reduction neutralisation titre, *PRNT50* 50% plaque reduction neutralisation titre, *FcγRIIIa* Fc gamma receptor IIIa

### Adolescent CD8^+^ T cell responses are boosted post-dose 3 of BNT162b2

**Fig. 2:**
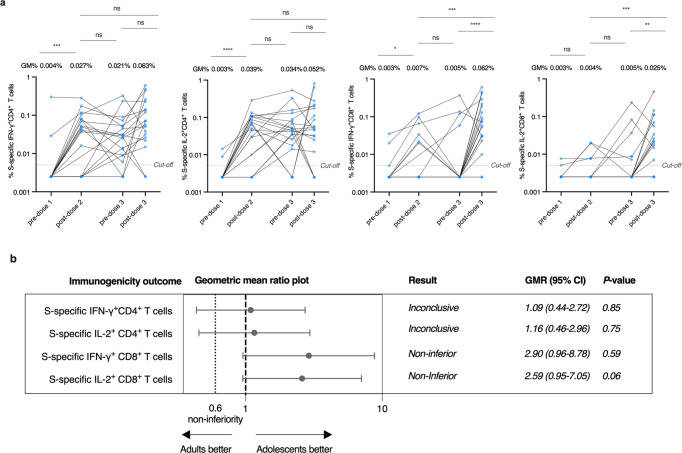
Adolescents have boosted CD8^+^ T cell responses to the WT virus after the third dose of the BNT162b2 vaccine. **a** Longitudinal analysis of S-specific interferon-γ (IFN-γ)^+^ CD4^+^, interleukin-2 (IL-2)^+^ CD4^+^, IFN-γ^+^ CD8^+^, and IL-2^+^ CD8^+^ T cells responses in evaluable adolescents (pre-dose 1 *N* = 18, post-dose 2 *N* = 18, pre-dose 3 *N* = 21, post-dose 3 *N* = 19). A third dose booster increased the S-specific IFN-γ^+^ CD8^+^ and IL-2^+^ CD8^+^ T cell responses, while IL-2^+^ CD8^+^ T cell responses were higher post-dose 3 compared to post-dose 2. Longitudinal analysis was determined using paired *t*-test after natural logarithmic transformation with *p* values denoted. Dots representing the same participants are linked by a straight line. Data labels and centre lines show geometric means (GM) estimates, with corresponding 95% confidence intervals shown by error bars. **b** Non-inferiority test of S-specific IFN-γ^+^ CD4^+^ (adolescent *N* = 26, adult *N* = 28), IL-2^+^ CD4^+^ (adolescent *N* = 26, adult *N* = 28), IFN-γ^+^ CD8^+^ (adolescent *N* = 26, adult *N* = 28) and IL-2^+^ CD8^+^ (adolescent *N* = 26, adult *N* = 28) T cell responses in evaluable adolescents in comparison to adults. Geometric mean ratios (GMR) and two-tailed 95% confidence intervals (CI) were plotted. S-specific IFN-γ^+^ CD8^+^ and S-specific IL-2^+^ CD8^+^ T cell responses were non-inferior, while S-specific IFN-γ^+^ CD4^+^ and S-specific IL-2^+^ CD4^+^ T cell responses were non-conclusive. Geometric mean ratios and their associated two-sided 95% confidence intervals (CIs) were plotted. *p < 0.05, **p < 0.01, ***p < 0.001, ****p < 0.0001. ns not significant. Cut-offs were drawn as grey lines. WT wild-type

IFN-γ^+^ and IL-2^+^ CD4^+^ and CD8^+^ T cells responses to SARS-CoV-2 overlapping S peptide pools were analyzed by flow cytometry. Compared to post-dose 2, T cells responses, including S-specific IFN-γ^+^ and IL-2^+^ CD4^+^ and CD8^+^ T cells, were not significantly different at pre-dose 3 (Fig. 2a). However, S-specific IFN-γ^+^ and IL-2^+^ CD8^+^ T cells increased significantly, with a respective 12.4-fold and fivefold increase at post-dose 3 when compared to that at pre-dose 3 (Fig. 2a). The increased S-specific IFN-γ^+^ and IL-2^+^ CD8^+^ T cell responses at post-dose 3 could also be detected in adults (Supplementary Fig. 3a).

A similar proportion of positive participants for WT S-specific IFN-γ^+^ (88.5 vs 82.1%) and IL-2^+^ (80.8 vs 78.6%) CD4^+^ T cells responses at a cut-off of 0.005% were detected in adolescents and adults after the third dose (Table 2). Interestingly, an increased proportion of positive participants for WT S-specific IFN-γ^+^ (84.6 vs 42.9%, *p* = 0.002) and IL-2^+^ (76.9 vs 50.0%, *p* = 0.052) CD8^+^ T cells responses were found in adolescents when compared to that in adults (Table 2). This result was further confirmed in the expanded analysis population (Supplementary Table 3). We also found all adolescents tested had a positive T cell response with at least one cytokine and subset, while that of adults was significantly lower (85.7%, *p* = 0.011) (Table 2). We also calculated the geometric mean ratio for T cell responses in adolescents versus adults (Fig. 2b). Comparisons of S-specific IFN-γ^+^ and IL-2^+^ CD4^+^ T cell responses to adults were inconclusive as the 95% CI limits were wide and beyond the non-inferiority margin of 0.60 and 1 (Fig. 2b). However, S-specific IFN-γ^+^ (GMR 2.90, 95% CI 0.96–8.78) and IL-2^+^ (GMR 2.59, 95% CI 0.95–7.05) CD8^+^ responses were non-inferior in adolescents compared to that in adults as the lower bounds of their two-sided 95% CI were above 0.60 (Fig. 2b and Supplementary Fig. 3b). The inconclusive S-specific IFN-γ^+^ and IL-2^+^ CD4^+^ T cell responses, but non-inferiority of S-specific IFN-γ^+^ and IL-2^+^ CD8^+^ responses in evaluable adolescents were further confirmed with that in expanded analysis populations (Supplementary Fig. 3c). These results show that the third dose of BNT162b2 induces potent cellular responses in adolescents, comparable to those in adults.

**Table 2:** Cellular immunogenicity outcomes against wild-type SARS-CoV-2 after the third dose of BNT162b2 in the evaluable analysis population

	Adolescents three doses	Adults three doses
**T cell responses**		
**S-specific T cell responses on flow cytometry**		
*N*	26	28
**GM** % IFN-γ^+^CD4^+^ T cells (95% CI)	0.058% (0.031–0.111%)	0.054% (0.027–0.106%)
% positive (≥cut-off at 0.01%)	88.5%, *P* = 0.71	82.1%
**GM** % IL-2^+^CD4^+^ T cells (95% CI)	0.046% (0.022-0.095%)	0.040% (0.021–0.075%)
% positive (≥cut-off at 0.01%)	80.8%, *P* > 0.9999	78.6%
**GM** % IFN-γ^+^CD8^+^ T cells (95% CI)	0.045% (0.023–0.091%)	0.016% (0.006–0.038%)
% positive (≥cut-off at 0.01%)	84.6%, *P* = 0.002	42.9%
**GM** % IL-2^+^CD8^+^ T cells (95% CI)	0.027% (0.012–0.059%)	0.010% (0.005–0.020%)
% positive (≥cut-off at 0.01%)	76.9%, *P* = 0.052	50.0%
% positive with at least 1 cytokine and subset (IFN-γ^+^/IL-2^+^CD4/8^+^)	100%, *P* = 0.011	85.7%

*P* values compare the proportion of positive responses between adolescents and adults by Fisher’s exact test

*S* Spike, *GM* geometric mean, *CI* confidence interval, *IFN-γ* interferon-gamma, *IL-2* interleukin-2

### Humoral and cellular immunity is maintained against Omicron after the third dose in adolescents and adults

**Fig. 3:**
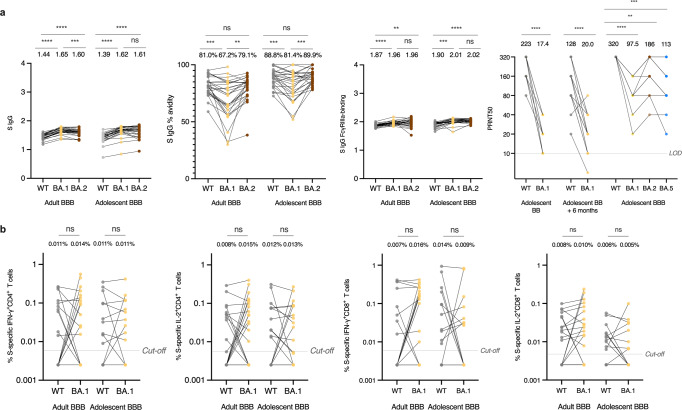
Humoral and cellular immunity is maintained against WT, Omicron BA.1 or BA.2, or BA.5 after the third dose of the BNT162b2 (BBB) vaccine in healthy evaluable adolescents and adults. **a** WT, BA.1, BA.2 and BA.5 SARS-CoV-2 Spike (S) IgG (Adult BBB *N* = 28, Adolescent BBB *N* = 28), S IgG avidity (Adult BBB *N* = 28, Adolescent BBB *N* = 28) and S IgG FcγRIIIa-binding (Adult BBB *N* = 28, Adolescent BBB *N* = 28) and PRNT50 (Adolescent BB *N* = 25, Adolescent BB + 6 months *N* = 25, Adolescent BBB *N* = 14) in evaluable adolescents and adults. Although antibody levels were quantitatively higher for BA.1 or BA.2 than WT, neutralisation was lower for BA.1, BA.2 or BA.5 than WT. **b** Omicron S WT reference pool and BA.1 mutation pool-specific interferon-γ (IFN-γ)^+^ CD4^+^ (Adult BBB *N* = 28, Adolescent BBB *N* = 22), interleukin-2 (IL-2)^+^ CD4^+^ (Adult BBB *N* = 28, Adolescent BBB *N* = 22), IFN-γ^+^ CD8^+^ (Adult BBB *N* = 28, Adolescent BBB *N* = 22), and IL-2^+^ CD8^+^ (Adult BBB *N* = 28, Adolescent BBB *N* = 22) T cells in evaluable adolescents and adults. The cellular responses were similar between WT and BA.1. Data labels and centre lines show geometric means (GM) estimates, with corresponding 95% confidence intervals shown by error bars. Dots representing the same participants are linked by a straight line. Statistical analysis was determined using paired *t*-test after natural logarithmic transformation with *p* values denoted. **p < 0.01, ***p < 0.001, ****p < 0.0001. ns not significant. LOD limit of detection, WT wild-type, FcγRIIIa Fcγ receptor IIIa, PRNT plaque reduction neutralisation test. Cut-offs were drawn as grey lines

We also sought to understand whether the third dose of BNT162b2 had increased immune responses against Omicron BA.1, BA.2 and/or BA.5 in adolescents. Omicron-specific binding antibody responses including Omicron BA.1 and BA.2-S IgG binding, IgG avidity and FcγRIIIa-binding antibodies, and BA.1, BA.2 and/or BA.5-neutralising antibody as measured by PRNT50 compared those to WT were compared in adolescents and adults. Interestingly, both S IgG and S FcγRIIIa-binding were conserved against both BA.1 and BA.2 after the third dose in evaluable adolescents when compared to those against WT, with marginally but significantly increased OD values (Fig. 3a). However, when compared to S IgG avidity against WT, it dramatically declined against BA.1, but was comparable to that against BA.2 both in adolescents and adults (Fig. 3a). 50% PRNT against BA.1, BA.2 and BA.5 were significantly lower than that of WT, yet remained detectable at moderate levels in all adolescents tested after 3 doses (Fig. 3a).

To investigate whether Omicron BA.1 variant could escape T cell recognition after the third dose in adolescents, an S mutation pool which contained peptides covering 37 BA.1-associated mutations was used. T cell responses were compared to those from the WT reference peptide pool. As expected, there were no significant differences between WT and BA.1 S-specific IFN-γ^+^ and IL-2^+^ CD4^+^ and CD8^+^ T cells responses in both adolescents and adults (Fig. 3b). These results indicate that the third dose of BNT162b2 elicits potent protection against Omicron subvariants both in adolescents and adults.

## Discussion

This study is the first to evaluate a wide range of humoral and cellular outcomes following a third dose of BNT162b2 in adolescents aged 11–17 years. A third dose can significantly boost antibody responses and CD8^+^ T cell responses in adolescents. These responses are similar compared to adults. Importantly, a third dose of BNT162b2 can provide cross-binding and neutralising responses to Omicron subvariants, and cross-reactive cellular responses to BA.1.

A high level of protection against SARS-CoV-2 infection and hospitalisation after two doses of mRNA vaccine in adolescents was found both in clinical trials and real-world data.^26,27^ Our previous data also showed boosting of humoral and cellular responses in adolescents after two doses compared with one dose.^8^ A third dose boosts the waning antibody response in adolescents. Similar to adult data, we showed reductions in neutralising antibodies, S IgG and FcγR-binding antibodies at 6 months after two doses of vaccine.^9^ However, in almost all parameters tested, the third dose of BNT162b2 was able to re-establish and enhance antibody responses. IgG avidity is used to measure the strength of binding of the S IgG response and is indicative of the formation of germinal centre reactions and high quality antibodies.^28,29^ Increasing IgG avidity over time correlates with the establishment of long-lasting spike antibody responses after both two and three doses. The non-inferior and superior S IgG avidity in adolescents compared to adults suggests that there may be more long-lasting antibodies generated in adolescents. However, a longer term follow-up will be required to observe whether these boosted S IgG, neutralising and high avidity responses will be maintained in adolescents. The FcγRIIIa-binding responses are the only serological response that remained static following the third dose in adolescents. FcγRIIIa-binding antibodies are associated with antibody-dependent cell cytotoxicity, clearance of immune complexes and killing of infected cells, thereby contributing to protection from severity during breakthrough Omicron infections.^30,31^ These FcγR antibodies increased significantly in adolescents and adults following two doses of BNT162b2^8^ but had not been assessed following three doses in adults or adolescents. Here we found that this potential correlate of protection does not increase further following the third dose in adolescents, and the response was non-inferior compared to adults.

Maintenance of S-specific CD4^+^ T cell responses at 6 months after two doses of vaccine is promising for the longevity of T cell responses in adolescents. The cross-reactive nature of T cell responses^32,33^ and their maintained responses against S observed here suggest long-lasting protection against future related variants in adolescents and adults. The lack of boosting in CD4^+^ T cell responses after the third dose of BNT162b2 was consistent with previous studies in adults^34^, which may be due to an immune ceiling being reached by significantly boosted and maintained responses following two doses. However, we observed a significant boost and enhancement in CD8^+^ T cell responses post-dose 3 in both adolescents and adults, although it was not seen in adults in previous studies.^34^ This lack of BNT162b2 significant boosting of T cell responses after two doses was seen in some adult cohorts^35^ but not others^36^ might be related to small sample sizes, assay sensitivity, or the differences in HLA in different geographic locations that led to variable epitope presentation, and therefore, variable responses.

Decreased vaccine efficacy of two doses alongside the surge of breakthrough infections with the Delta variant (B.1.617.2) and Omicron BA.1 of SARS-CoV-2 prompted the rapid rollout of the third dose of BNT162b2 vaccine globally.^14^ Omicron BA.1 was first reported to the WHO by South Africa on November 24, 2021,^37^ and caused significant concern due to a large number of mutations, especially in the Spike protein. Many studies in healthy adults have reported the dramatic escape of neutralisation antibodies by Omicron variants after two doses, but increased antibody-based immunity against Omicron after three doses of BNT162b2.^37,38^ In healthy adults from another cohort, using the same assay, we found 50% PRNT GMT of 67.3 and 95.1 against BA.1 and BA.2 3 weeks after 3 doses.^38,39^ In contrast, our data showed that adolescents had higher GMT against BA.2 after three doses (GMT 186), which was comparable to PRNT50 against WT after 2 doses of BNT162b2 in adults. This result is also consistent to our findings between BA.2 and WT in terms of S IgG avidity. We also noted that S IgG avidity was significantly higher with BA.2 than BA.1, which may be due to BA.1 having 39 mutations and BA.2 only having 32 mutations in S compared to WT as additional mutations in BA.1 S might impair antibody avidity. Although neutralisation of BA.1, BA.2 and BA.5 was significantly lower than for WT after 3 doses in adolescents, it was still detectable at a moderate level (GMT for BA.1 97.5, BA.2 186 and BA.5 113), similar to PRNT50 against WT after WT SARS-CoV-2 infection in adults.^38,40^ Our data also showed that adolescents had preserved levels of binding antibodies as measured by S IgG and S IgG FcγRIIIa-binding antibodies against BA.1 and BA.2 after the third dose of BNT162b2 when compared with that against WT. Indeed, another study also showed that Omicron S-specific binding for IgG and FcγRIIIa persisted at a high level across the two doses of mRNA vaccine.^41^ Besides neutralising antibodies, the non-neutralising antibodies like S IgG FcγRIIIa-binding can lead to continued viral clearance and the killing of infected cells, finally, may contribute to the less severe Omicron infection.^30,31^ Therefore, our results demonstrate that the third dose of BNT162b2 can provide protection against Omicron subvariants by potent cross-reactive binding and neutralising antibody and T cell responses.

There are limitations to this study. First, we compared humoral and cellular responses after the third dose of the BNT162b2 vaccine on a limited subset of samples due to the surge of breakthrough infections with the Omicron BA.2 of the SARS-CoV-2 in Hong Kong during the study period, when some participants were infected or defaulted follow-up clinic to avoid potential Omicron BA.2 transmission. Limitation in blood volume obtained from adolescents precluded us from detecting small differences in some outcomes between adults and adolescents, yet most of our outcomes tested satisfied non-inferiority testing, demonstrating that diverse immune responses in adults and adolescents were not different by a clinically significant margin. Because of the critical roles of T helper (Th) 1 in virus controlling^42^ and the limitation of blood volume obtained from adolescents, we focused our efforts on quantifying post-vaccine Th1 T cell responses. Previously, in an adult vaccine study, we found that Spike-specific IL-4^+^ Th2 responses are not boosted by two-dose BNT162b2 vaccination, and the majority of post-vaccine responses were IFN-γ^+^ T effector memory responses.^35^ However, at SARS-CoV-2 infection, age-dependent differences are seen in T cell responses magnitude and phenotype for Th1, Th2, activated cells, increased total Tfh responses and reduced effector memory CD4^+^ T cells in young children.^43^ Our previous work in adolescents versus adults did not find a difference in post-vaccine T cell responses for either Coronavac and BNTb162 at two doses of vaccination.^8^ Therefore, further work at vaccination to determine T cell phenotype may reveal differences in T cell recall potential. We did not investigate every Omicron subvariant at each timepoint due to assay availability. Hybrid immunity, whether by vaccinating infected adolescents or breakthrough infections in vaccinated adolescents, was not investigated in our study, but more studies on this will be important for understanding the long-term immunological implications of vaccination in this population.^44–46^

Taken together, our data suggest cross-reactive potent antibody and T cell responses are elicited by a third dose of BNT162b2 in adolescents, explaining the high vaccine effectiveness observed in real-world studies. We will further track the durability of immunogenicity after three doses of the BNT162b2 vaccine and hybrid immunity after breakthrough infections.

## Materials and methods

### Study design

Coronavirus disease-19 (COVID-19) Vaccination in Adolescents and Children (COVAC; NCT04800133) aimed at evaluating the humoral and cellular immunogenicity in children.^8^ This study was approved by the University of Hong Kong (HKU)/Hospital Authority Hong Kong West Cluster Institutional Review Board (UW21-157).

### Participants

This study included healthy adolescents aged 11–17 years and adults aged 18 years or older who received three doses of BNT162b2 intramuscularly. Potential participants with stably healthy conditions, a known history of COVID-19, a history of severe allergy, significant neuropsychiatric conditions, immunocompromised states were included. Transfusion of blood products within 60 days, haemophilia, pregnancy or breastfeeding were excluded from this study.

### Procedures

Potential participants were recruited via school, media, or referral in Hong Kong. Study physicians contacted and obtained informed consent from participants aged 18 years or above, or for underage participants, from their parents and legally acceptable representatives.

### S-RBD, surrogate virus neutralisation assay (sVNT) and plaque reduction neutralisation test (PRNT)

Peripheral clotted blood was drawn, and the serum was stored at −80 °C after separation. The SARS-CoV-2 S receptor-binding domain (R-SBD) IgG enzyme-linked immunosorbent assay (ELISA) and PRNT were carried out as previously described and validated.^8^ sVNT was conducted according to the manufacturer’s instructions (GenScript Inc, Piscataway, USA) and as described in our previous publication. All sera were heat-inactivated at 56 °C for 30 min (mins) before testing.^47,48^ Details for the detections of S-RBD IgG, sVNT and PRNT were performed by the same methods shown in our previous study.^8^ Briefly, S-RBD IgG ELISA plates were coated overnight with 100 ng/well of purified recombinant S-RBD in PBS buffer, followed by the incubation with 100 µL Chonblock Blocking/Sample Dilution (CBSD) ELISA buffer (Chondrex Inc, Redmond, USA) at room temperature (RT) for 2 h. Then added, the 1:100 diluted serum in CBSD ELISA buffer to the wells and incubated at 37 °C for another 2 h. After washing with 0.1% Tween 20 PBS (PBST), the plates were incubated with 1:5000 diluted horseradish peroxidase (HRP)-conjugated goat anti-human IgG (Thermo Fisher Scientific) at 37 °C for 1 h and washed with PBST for five times. Finally, 100 µL of HRP substrate (Ncm TMB one, New Cell & Molecular Biotech. Ltd. China) was added for 15 min before stopping this reaction by 50 µL of 2 M H_2_SO_4_. The optical density (OD) was analyzed in a Sunrise absorbance microplate reader (Tecan, Männedorf, Switzerland) at 450 nm wavelength. Each OD reading was calculated by subtracting the background OD in PBS-coated control wells from the serum of participants. Values at or above an OD450 of 0.5 were considered positive, otherwise were imputed as 0.25.

For sVNT detection, 10 µL of serum were diluted at 1:10 and incubated with an equal volume HRP conjugated to the WT SARS-CoV-2 S-RBD (6 ng) at 37 °C for 30 min, followed by the addition of 100 µL of each sample to each well of microtitre plates coated with angiotensin-converting enzyme-2 (ACE-2) receptor at 37 °C for 15 min. After washing and drying, 100 µL of 3,3′,5,5′-tetramethylbenzidine (TMB) was added and incubated at RT far away from light for 15 min. Finally, the reaction was terminated, and the absorbance was read at 450 nm in a microplate reader. After confirmation that the positive and negative controls provided the recommended OD450 values, the % inhibition of each serum was calculated as (1 − sample OD value/negative control OD value) × 100%. Inhibition (%) of at least 30%, the limit of quantification (LOQ), was regarded as positive, while values below 30% were imputed as 15%.

The PRNT assay was performed in duplicate under a facility with biosafety level 3 as described before.^8^ In brief, serum was diluted from 1:10 to 1:320, and then incubated with BetaCoV/Hong Kong/VM20001061/2020 (WT strain), hCoV-19/Hong Kong/ VM21044713_WHP5047-S5/2021 (Omicron BA.1), hCoV-19/Hong Kong/VM22000135_HKUVOC0588P2/2022 (Omicron BA.2), or SARS-CoV-2/human/USA/COR-22-063113/2022 (Omicron BA.5) at 30 plaque-forming units in a culture plate (Techno Plastic Products AG, Trasadingen. Switzerland) at 37 °C for 1 h. Then the virus-serum mixtures were added to Vero E6 TMPRESS2 cell monolayers and further incubated at 37 °C for 1 h. The plates were overlaid with 1% agarose in a cell culture medium and incubated for 3 days. After fixing and staining, antibody titres were defined as the reciprocal of the highest serum dilution that resulted in ≥90% (PRNT90, a more stringent cut-off) or >50% (PRNT50) reduction in the number of plaques. Values below the lowest dilution tested of 10 were imputed as 5.

### S IgG, avidity and FcγRIIIa-binding

Detections of S IgG, avidity and FcγRIIIa-binding were carried out as previously described.^8^ Briefly, proteins were diluted in PBS for specific antibody detection. Firstly, Plates (Nunc MaxiSorp., Thermo Fisher Scientific) were coated with 250 ng/mL WT (AcroBiosystems) or Omicron BA.1 (AcroBiosystems) or Omicron BA.2 (AcroBiosystems) SARS-CoV-2 S protein for IgG and IgG avidity detections, or 500 ng/mL WT (Sinobiological) or Omicron BA.1 (AcroBiosystems) S for FcγRIIIa-binding detections, or 300 ng/mL ORF8 (Masashi Mori, Ishiwaka University, Japan) at 37 °C for 2 h.^25,49^ The detection of ORF8-specific IgG was used to exclude infected individuals.

For IgG detection, plates were blocked with 1% FBS in PBS for 1 h before incubating with heat-inactivated (HI) serum, which was 1:100 diluted in 0.05% Tween 20/0.1% FBS in PBS at RT for 2 h. For antibody avidity, plates were washed three times with 8 M Urea before incubating with IgG-HRP (1:5000, G8-185, BD) for 2 h. HRP was revealed by stabilised hydrogen peroxide and tetramethylbenzidine (R&D systems) for 20 min, then stopped with 2 N H_2_SO_4_ and analysed with an absorbance microplate reader at 450 nm wavelength (Tecan Life Sciences). For FcγRIIIa-binding measurement, plates were coated with 500 ng/mL S protein and incubated with 1:50 diluted HI serum at 37 °C for 1 h before incubation with 100 ng/mL biotinylated FcγRIIIa-V158 at 37 °C for 1 h, followed by the detection of S-specific FcγRIIIa-V158-binding antibodies by using streptavidin-HRP (1:10000, Pierce). OD values in ELISA-based antibody tests were normalised across experiments using WHO international standards and calibrated internal standards.^50^

### T cell responses

Peripheral blood mononuclear cells (PBMCs) were isolated from the whole blood of participants by density gradient separation and stored in liquid nitrogen before use. Firstly, PBMCs were thawed in RPMI medium supplemented with 10% human AB serum, then rested in a 37 °C incubator for 2 h. The cells were stimulated with 1 µg/mL overlapping peptide pools representing the WT SARS-CoV-S proteins (Miltenyi Biotec, Bergisch Gladbach, Germany), or B.1.1.529/BA.1 S mutation pool (Miltenyi Biotec, Bergisch Gladbach, Germany) and WT reference pool (Miltenyi Biotec, Bergisch Gladbach, Germany), supplemented with 1 µg/mL anti-CD28 and anti-CD49d costimulatory antibodies (Clones CD28.2 and 9F10, respectively, Biolegend, San Diego, USA) at 37°C for 16 h. An equal volume of sterile double-distilled water (ddH2O) was used as a negative control. This mixture was stimulated for 2 h, followed by the addition of Brefeldin A (BFA,10 µg/mL; Sigma, Kawasaki, Japan).^51^ Secondly, the cells were washed and immunostained with a fixable viability dye (eBioscience, Santa Clara, USA, 1:60), and antibodies against CD3 (HIT3a, 1:60), CD4 (OKT4, 1:60), CD8 (HIT8a, 1:60), followed by fixed, permeabilized and stained with antibodies against IFN-γ (B27, 1:15) and IL-2 (MQ1-17H12, 1:15). All of these antibodies were purchased from Biolegend. Finally, data acquisition was carried out using flow cytometry (LSR II, BD Biosciences, Franklin Lakes, USA) and analyzed by FlowJo v10 software (BD, Ashland, USA). Supplementary Fig. 4 shows the gating strategies for CD4^+^ and CD8^+^ T cell analysis and representative flow cytometry plots for negative and positive controls, and peptide-stimulated PBMCs. Antigen-specific IFN-γ^+^ and IL-2^+^ T cell results were finalised after subtracting the background (ddH2O) data and presented as the percentage of CD4^+^ or CD8^+^ T cells.^52^ T cell responses against a single peptide pool were considered positive when the frequency of cytokine-expressing cells was higher than 0.005% and the stimulation index was higher than 2, while negative values were imputed as 0.0025%.

### Outcomes

Humoral immunogenicity (S IgG and S-RBD IgG levels, sVNT %inhibition, 90% and 50% PRNT titres, S IgG avidity and FcγRIIIa-binding) and cellular immunogenicity markers (S-, Omicron S mutation- and Omicron WT reference- specific IFN-γ^+^ and IL-2^+^ CD4^+^ and CD8^+^ T cell responses) assessed after the third dose of BNT162b2.

### Statistical analyses

#### Sample size

As the study was conducted during the Omicron BA.2 wave in Hong Kong, participants who were infected were excluded and some participants defaulted to vaccination or follow-up clinic. All evaluable samples were tested by S-RBD IgG and sVNT, and sample sizes for more demanding assays, e.g. PRNT and T cell testing, were reduced based on laboratory capacity.

#### Analysis sets

The primary analysis of humoral and cellular immunogenicity outcomes was performed in the healthy adolescents and adults in the evaluable analysis population who received an intramuscular injection of the BNT162b2 vaccine on a per-protocol basis, as described before.^8^ All of these evaluable populations remained uninfected during study visits based on self-reporting, ORF8 IgG negativity and negative baseline S-RBD IgG, had no major protocol deviations. Each immunogenicity outcome was calculated by GM, and GM ratios (GMRs) were reported with a two-sided 95% CI, corresponding to a one-sided 97.5% CI, to test the non-inferiority hypothesis at the margin of 0.60. Non-inferiority analyses were further confirmed in the expanded analysis population (Supplementary Table 4). The blood sampling intervals were chosen based on previous publications and guideline recommendations.^8,53^ When both non-inferiority and inferiority were not met, the results were inconclusive. Participants with valid results at consecutive timepoints were compared by GM fold rise (GMFR). Immunogenicity outcomes data below the cut-off were imputed with half the cut-off value. Immunogenicity outcomes were analysed by an unpaired or paired *t*-test after natural logarithmic transformation. The proportion of participants with a positive result was reported in percent with 95% CI derived from the Clopper–Pearson method. The comparisons of proportions between groups were performed with the Fisher exact test.

## Supplementary information

supplementary materials

## Data Availability

The data used in the current study are available from the corresponding authors upon reasonable request.
